# Innovating Clinical Trials for Amyotrophic Lateral Sclerosis: Challenging the Established Order

**DOI:** 10.1212/WNL.0000000000012545

**Published:** 2021-09-14

**Authors:** Ruben P.A. van Eijk, Stavros Nikolakopoulos, Kit C.B. Roes, Lindsay Kendall, Steve S. Han, Arseniy Lavrov, Noam Epstein, Tessa Kliest, Adriaan D. de Jongh, Henk-Jan Westeneng, Ammar Al-Chalabi, Philip Van Damme, Orla Hardiman, Pamela J. Shaw, Christopher J. McDermott, Marinus J.C. Eijkemans, Leonard H. van den Berg

**Affiliations:** From the Department of Neurology, UMC Utrecht Brain Center (R.P.A.v.E., T.K., A.D.d.J., H.-J.W., L.H.v.d.B.), and Biostatistics & Research Support, Julius Center for Health Sciences and Primary Care (R.P.A.E., S.N., M.J.C.E.), University Medical Center Utrecht; Department of Health Evidence (K.C.B.R.), Section Biostatistics, Radboud Medical Centre Nijmegen, the Netherlands; Biostatistics (L.K.), GlaxoSmithKline R&D, Stevenage, UK; Neurosciences (S.S.H.), Takeda Pharmaceuticals, Cambridge, MA; Discovery Medicine (S.S.H., N.E.), GlaxoSmithKline R&D, Upper Providence, PA; Clinical Development (A.L.), Novartis Gene Therapies; Clinical Translational Medicine (A.L.), Future Pipeline Discovery, GlaxoSmithKline R&D, Middlesex; Maurice Wohl Clinical Neuroscience Institute and United Kingdom Dementia Research Institute Centre, Department of Basic and Clinical Neuroscience, King's College London (A.A.-C.); Department of Neurology (A.A.-C.), King's College Hospital, London, UK; Department of Neurosciences (P.V.D.), Laboratory for Neurobiology, KU Leuven and Center for Brain & Disease Research, VIB, Leuven Brain Institute; Department of Neurology (P.V.D.), University Hospitals Leuven, Belgium; Department of Neurology (O.H.), National Neuroscience Centre, Beaumont Hospital, Dublin, Ireland; FutureNeuro SFI Research Centre (O.H.), Royal College of Surgeons in Ireland, Dublin; and Department of Neuroscience (P.J.S., C.J.M.), Sheffield Institute for Translational Neuroscience, University of Sheffield, UK.

## Abstract

Development of effective treatments for amyotrophic lateral sclerosis (ALS) has been hampered by disease heterogeneity, a limited understanding of underlying pathophysiology, and methodologic design challenges. We have evaluated 2 major themes in the design of pivotal, phase 3 clinical trials for ALS—(1) patient selection and (2) analytical strategy—and discussed potential solutions with the European Medicines Agency. Several design considerations were assessed using data from 5 placebo-controlled clinical trials (n = 988), 4 population-based cohorts (n = 5,100), and 2,436 placebo-allocated patients from the Pooled Resource Open-Access ALS Clinical Trials (PRO-ACT) database. The validity of each proposed design modification was confirmed by means of simulation and illustrated for a hypothetical setting. Compared to classical trial design, the proposed design modifications reduce the sample size by 30.5% and placebo exposure time by 35.4%. By making use of prognostic survival models, one creates a potential to include a larger proportion of the population and maximize generalizability. We propose a flexible design framework that naturally adapts the trial duration when inaccurate assumptions are made at the design stage, such as enrollment or survival rate. In case of futility, the follow-up time is shortened and patient exposure to ineffective treatments or placebo is minimized. For diseases such as ALS, optimizing the use of resources, widening eligibility criteria, and minimizing exposure to futile treatments and placebo is critical to the development of effective treatments. Our proposed design modifications could circumvent important pitfalls and may serve as a blueprint for future clinical trials in this population.

Development of treatments for amyotrophic lateral sclerosis (ALS) has been challenging and, despite considerable efforts, only riluzole has been proven to prolong survival time by 2 to 3 months.^1-3^ Recently, the Treatment Research Initiative to Cure ALS has extended a collective effort among academia, patient advocacy groups, industry partners, and fundraisers to reform clinical trial design and to improve the likelihood of successful drug development.^4^

Over the years, a number of suggestions have been made to innovate the design of clinical trials in ALS.^1,3,5-7^ Nevertheless, clinical trials have remained relatively conservative, especially when initiated by industry, as deviating from trial guidelines could affect regulatory acceptability. Though industry is open to fundamentally changing drug development for ALS,^4^ they require amendments of the current regulatory guidelines to successfully adopt innovation in their pipelines.

In an attempt to address these concerns, we have evaluated 2 major themes in the design of pivotal, phase 3 clinical trials for ALS: (1) patient selection and (2) analytical strategy. Virtually all clinical trials impose various sets of eligibility criteria to reduce clinical heterogeneity.^5,8^ This leads to the exclusion of many patients, which affects generalizability (i.e., “to whom do the results of this trial apply?”).^9^ Moreover, such criteria have been minimally effective in creating more homogenous trial populations.^5^ In addition, funding and resources for low prevalence disorders such as ALS are limited, while costs of pivotal studies are high, requiring relatively large sample sizes and long follow-up periods to provide confirmatory evidence.^10^

To address these challenges, we illustrate the rationale for innovative design modifications in clinical trials for ALS. Our considerations were discussed with the European Medicines Agency (EMA) during a scientific advice session on June 25, 2020, and are illustrated using patient-level data from 5 placebo-controlled clinical trials (n = 988, including active and placebo patients due to lack of efficacy),^11-15^ 4 population-based incidence cohorts (n = 5,100; Leuven, Sheffield, Dublin, and Utrecht), and 2,436 placebo-allocated patients from the Pooled Resource Open-Access ALS Clinical Trials (PRO-ACT) database.^5^

## Standard Protocol Approvals, Registrations, and Patient Consents

The medical ethics committee and institutional review board of the University Medical Center Utrecht approved reanalysis of the data for the purposes outlined in this work.

## Data Availability

All analyses and anonymized data will be shared by request from any qualified investigator.

## Refining Patient Eligibility: Risk-Based Patient Selection

Among the first design considerations for clinical trials are the inclusion and exclusion criteria. Depending on the study phase, selection criteria may aim to enroll a more homogeneous population, improve protocol adherence and safety, or exclude patients who are unlikely to benefit from treatment.^8,9,16^ Trial eligibility is classically determined by a stepwise assessment of patient characteristics such as age or disease severity. To illustrate: trial X aims to reduce dropout and selects patients younger than 75 years with a vital capacity (VC) of ≥60%. However, fulfilling these criteria does not guarantee that the patient completes the study. For example, a patient who fails one of the criteria (e.g., patient 1, 76 years, VC 100%) may have a higher probability of completing the study as compared to a patient fulfilling all criteria (e.g., patient 2, 74 years, VC 61%). This phenomenon is explained by the fact that progression rate, prognosis, and dropout are dictated by a multivariable combination of characteristics.^5,17,18^ In contrast, stepwise criteria evaluate patient characteristics univariably and ignore their combined effect on prognosis. The accuracy of patient enrollment can thus be improved by evaluating multiple characteristics simultaneously by, for example, making use of multivariable prediction models.^5^

### Risk Profile as Prognostic Summary

Prediction models are a method for summarizing multiple patient characteristics into an individual risk profile. This “prognostic summary” could serve as a targeted approach to exclude patients with an undesirable prognosis and maximize eligibility rates (i.e., the number of patients who fulfil the enrolment criteria).^5,6^ Here we illustrate patient eligibility based on their (relative) survival risk profiles as estimated by the cross-validated European Network for the Cure of ALS (ENCALS) survival model.^17^ Nevertheless, other prediction rules have been proposed and may be used in a similar fashion.^19^ The ENCALS risk profile, hereafter referred to as “risk profile,” is a weighted average of patient characteristics; its calculation is available from Dryad (eAppendix 1, doi.org/10.5061/dryad.fbg79cnv7).

**Figure 1:**
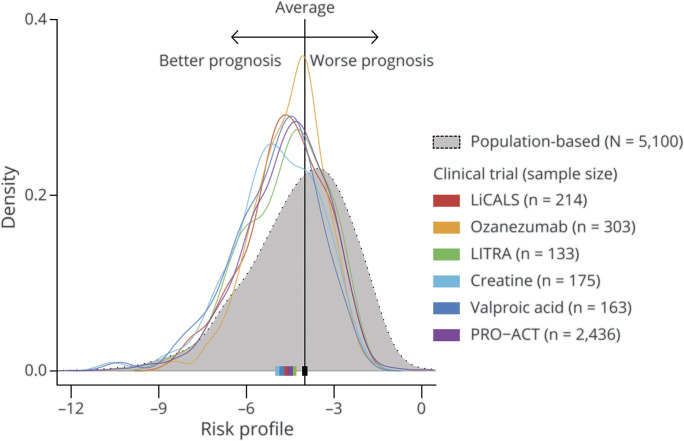
Distribution of Risk Profiles in Clinical Trial and Population-Based Cohorts For each patient, we calculated his or her risk profile (horizontal axis), a composite score of 7 prognostic variables, which ranges from approximately −12 (very long prognosis) to 0 (very short prognosis). The distribution of the risk profiles is given as density curves, with the probability density on the vertical axis. The interpretation of the figure is similar to that of a histogram. The colors represent different trial populations; the population-based cohort is in gray. The solid squares are the population medians. A clear shift is observed in trial populations towards a better prognosis compared to a population-based cohort, which reflects the underrepresentation of patients with a poor prognosis in clinical trials. Exact ranges per cohort are available from Dryad (eAppendix 1, doi.org/10.5061/dryad.fbg79cnv7); LiCALS = lithium carbonate in patients with amyotrophic lateral sclerosis; PRO-ACT = Pooled Resource Open-Access ALS Clinical Trials.

In population-based cohorts, the risk profile ranges approximately from −12.0 to 0.0, with higher scores indicating a worse prognosis. Figure 1 depicts the distribution of risk profiles for 6 clinical trial cohorts (n = 3,424) compared to a population-based incidence cohort (n = 5,100); the population ranges are available from Dryad (eAppendix 1, doi.org/10.5061/dryad.fbg79cnv7). The distribution of risk scores among clinical trial participants represents a more favorable prognosis compared to the population-based cohort. This translates to the increased survival of trial participants as compared to population-based datasets observed in previous studies.^5,8,20^ Notably, patients with a risk profile of ≥−2.0 (i.e., very short survivors) are virtually nonexistent in trial populations (<1.0%), while they account for 8.5% of the general ALS population. This observation is primarily driven by the delay between diagnosis and trial enrollment, a period in which most very short survivors die or become too weak to participate in a clinical trial.

### Predictive Properties of the Risk Profile for Disease Progression Rate

A key objective of inclusion criteria may be to remove nonprogressing or rapidly progressing patients as (1) most treatments will require some exposure time before any effect on clinical endpoints can be detected and (2) therapeutic effects may not be efficiently measured in nonprogressing patients and would increase sample size or require a longer trial.^21^ Previous studies have revealed the predictive properties of the risk profile for overall survival.^5,17^ Here we illustrate that among all clinical trials reported in figure 1, the rate of decline during follow-up was strongly related to the patient's risk profile at baseline (all *p* < 0.001; Table 1): with each unit increase in risk profile, the monthly rate of decline increased by 0.20 (95% confidence interval, 0.18–0.21) points per month. This observation may not be surprising given the predictive value of ALS Functional Rating Scale–Revised (ALSFRS-R) progression for survival time.^18^ Moreover, there is a natural relationship between a poor prognosis, a short survival time, and a fast disease progression rate. Selection based on the risk profile may therefore not only affect survival and functional decline during follow-up, but might also be used to replace stepwise criteria that aim to remove fast or slow progressing patients.

**Table 1:**
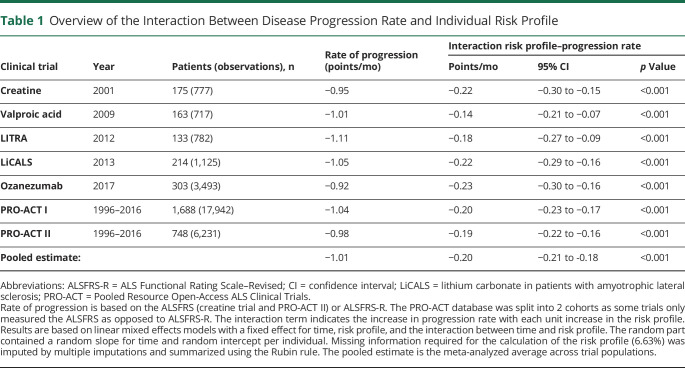
Overview of the Interaction Between Disease Progression Rate and Individual Risk Profile

### Risk-Based Patient Selection and Heterogeneity in Progression Rates

To illustrate its use as an inclusion criterion, one can define an eligibility window based on the risk profile, which can be determined pragmatically or driven by sample size. For example, 202 patients would be required to detect a 25% reduction in ALSFRS-R slope when enrolling patients with risk profiles between −4.0 and −2.0, whereas sample size increases to 262 (+30%) when widening the window to −6.0 to −2.0.^22^ The exclusion rates for each selection window are 59% and 24%, respectively. As comparison, utilizing classic criteria, for example, symptom duration ≤24 months, age ≤80 years, and VA ≥65%,^23^ would result in an exclusion rate of 35% and a required sample size of 278. Although this example reveals the beneficial effect of risk-based selection on the trial's sample size, a too narrow eligibility window may restrict the trial's generalizability and compromise enrollment rates. It may therefore be advisable to define a maximum exclusion rate (say 25%) and subsequently determine which eligibility window results in the smallest sample size. To illustrate this point, in Table 2 we provide the patient characteristics of eligible patients with a risk profile −6.0 to −2.0, resulting in an exclusion rate of less than 25%. The risk-based selection results in a similar excluded population that contains the patients who are commonly omitted from trials (e.g., long disease duration, low forced VC, or rapidly progressive ALS). Of note, the final enrolled population is not solely driven by eligibility, but could also be affected by latent processes, such as patient preference, cultural differences, or physician-related factors.

**Table 2:**
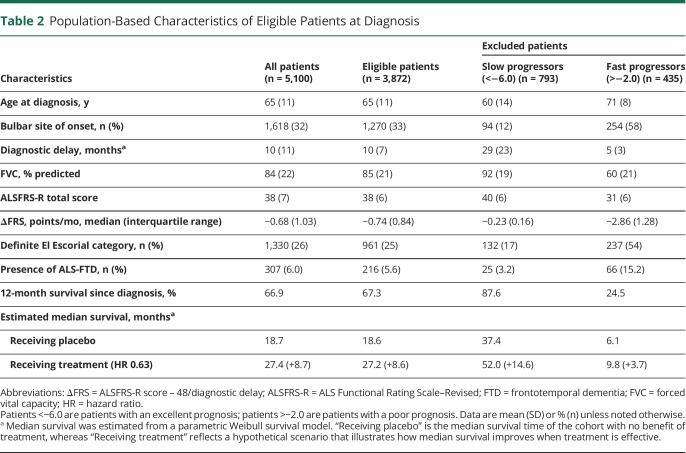
Population-Based Characteristics of Eligible Patients at Diagnosis

### Considerations and Refinement After Regulatory Feedback

As illustrated previously,^5^ the approach of using a risk profile as inclusion criterion may have potential to include a larger part of the population by better predicting prognosis, disease progression, and possibly even dropout. Nevertheless, defining the eligibility window must be carefully weighed against the reduction in sample size: can a minimal exclusion rate be achieved without a relevant increase in sample size? The definition of a relevant loss of trial patients, and the resulting optimal eligibility window, depends on the investigator preferences and the setting (e.g., broad vs genotype-targeted treatments, or in combination with additional biomarker criteria), and may need to be fine-tuned and substantiated on a study-by-study basis. The comparability of results across trials, or evaluating shifts in the population risk profile over time, may help to further refine the eligibility window. Finally, the performance of the model must be prospectively validated, in external populations, and investigators should evaluate the model performance in both included and excluded patients. During the conduct of the trial, it would be important to carefully register intercurrent events (e.g., change in riluzole intake during follow-up) and evaluate whether and how these affect the prediction accuracy of the model.

## Optimizing Analytical Design of Survival Outcomes

In current regulatory guidelines,^3,24^ an independent assessment of survival time is required to characterize efficacy in pivotal clinical trials. In the following sections, we will describe several key design considerations for ALS survival outcomes, illustrated for a hypothetical study. Because very short survivors are underrepresented in trial populations (Figure 1), the number of patients who have an event at the beginning of follow-up is considerably lower compared to the end of follow-up.^10^ All calculations and simulations in the following sections are therefore based on the Weibull framework, with an increasing hazard over time and an 18-month survival of 56.1% (based on the placebo patients in PRO-ACT with a risk profile between −6.0 and −2.0).^10,25^

### Optimizing Time Requirements: Extended Randomized Follow-up

Optimizing the use of time can considerably improve the power of survival outcomes to detect treatment responses.^26^ Because the number of events (e.g., deaths) drives their power,^27,28^ solely extending follow-up time, while keeping the sample size constant, can increase the power to detect treatment effects. An important consideration is therefore to extend the randomized follow-up for early-enrolled patients and, as a consequence, generate more events within the same timeframe.^10^ To illustrate: an 18-month trial (n = 300) with a 12-month enrollment period could extend the follow-up for early-enrolled patients, while retaining an 18-month follow-up for late-enrolled patients, increasing power from 68.5% to 84.6% to detect a hazard ratio (HR) of 0.63.^28,29^ The extended follow-up can be implemented with minimal effort and could result in important reductions in sample size and costs.^10,26^ From a patient perspective, the drawback of such an approach is the uncertainty about the length of trial participation and the potentially longer time on placebo if enrolled early. These limitations could be mitigated by implementing interim analyses (illustrated below), or by using a hybrid approach, with a maximum follow-up period for early enrolled patients. An example of the latter can be found in the dexpramipexole study.^23^

### Accuracy of a Priori Assumptions and Trial Design

Designing clinical trials with time-to-event endpoints requires a considerable number of assumptions; for example, we made assumptions above about the survival pattern (Weibull), survival probability (56.1%), and enrollment period (12 months). Despite the fact that these assumptions can be estimated using historical data, an inherent risk of inaccuracy remains. Making inaccurate a priori assumptions at the design stage (i.e., misspecification) can have detrimental consequences for a trial's ability to draw definite conclusions.^30,31^ If the observed 18-month survival is 50.0% or 60.0% instead of 56.1%, the required sample size fluctuates between 237 and 286 patients (a difference of up to 21%; data available from Dryad, eAppendix 1, doi.org/10.5061/dryad.fbg79cnv7). As a result, inaccuracy in design assumptions could significantly over- or underpower clinical trials and unnecessarily expose patients to harmful or ineffective treatments.

### Event-Driven Trial Design as Guard Against Misspecification

The required sample size for time-to-event outcomes is calculated as the required number of events divided by the probability of an event.^29^ The probability of an event depends on various assumptions (e.g., survival distribution, dropout rate, and enrollment period), whereas the number of events depends solely on the hypothesized treatment effect (HR), allocation ratio, power, and type 1 error.^27,28^ In other words, irrespective of the follow-up duration, survival distribution, or enrollment rate, as long as the required number of events is obtained, the trial will reach its designated power. This is a useful feature of time-to-event endpoints and it has been suggested, therefore, to run the trial until a prespecified number of events is reached (i.e., event-driven or information-based trials) rather than concluding the trial after a fixed duration of follow-up (i.e., duration-based trials).^32-34^ The major benefit of the event-driven approach is that the accuracy of the event probability becomes irrelevant. The major drawback is that the exact trial duration is unknown (i.e., when is the target number of events reached?) and cannot be fixed from the outset.^32^ Nevertheless, using various projections about the underlying assumptions, the uncertainty in trial duration can be estimated and used as a guide by investigators, or to inform patients about the expected individual follow-up time.

### Group-Sequential Interim Analyses

The last design consideration is to implement an interim analysis scheme to stop the trial early because of either inefficacy (i.e., futility or harm) or efficacy (i.e., superiority). Implementing interim analyses could considerably increase a trial's efficiency and reduce the exposure of patients to ineffective treatments or placebo.^7,35^ Similar to the final analysis, the timing of interim analyses can be guided by the number of events. Implementing an interim analysis scheme does, however, increase the required number of events to account for multiplicity. For example, incorporating a single interim analysis after the occurrence of 60% of the events requires 2.9% more events compared to a design without interim analyses (using a conservative O'Brien-Fleming type alpha-spending function).^36^ The expected number of events required to reach a conclusion is, however, 25.6% less if the treatment is futile, and 13.9% less if the treatment is superior. Thus, if a trial cannot be stopped early, slightly more events are needed, but on average fewer events are required to reach a conclusion compared to a design without interim analyses.^7^

### Illustration of Event-Driven, Group-Sequential Trial

Finally, by means of simulation, we will illustrate the above-mentioned design considerations for a hypothetical study. The study targets a total of 153 events to detect an HR of 0.63 with 80% power and a 1-sided α of 2.5%. In this setting, an HR of 0.63 translates to a median survival increase after enrollment from 19.7 to 24.8 months (+5.1 months); in Table 2, the median survival estimates are provided for other populations. A single interim analysis will occur after 60% of the required events. Assuming an enrollment period of 12 months, it can be estimated that 278 patients are required (139 active vs 139 placebo) to reach the target number of events within 30 months after first enrolled patient.^10^ The trial setting was simulated under both the null (i.e., HR 1) and the alternative (i.e., HR 0.63) hypothesis, where each simulated trial was run until an interim decision was reached or until 153 events occurred. Based on 100,000 simulations, the 1-sided type I error was 2.45%, whereas the empirical power was 80.4%, confirming the validity of the design.

**Figure 2:**
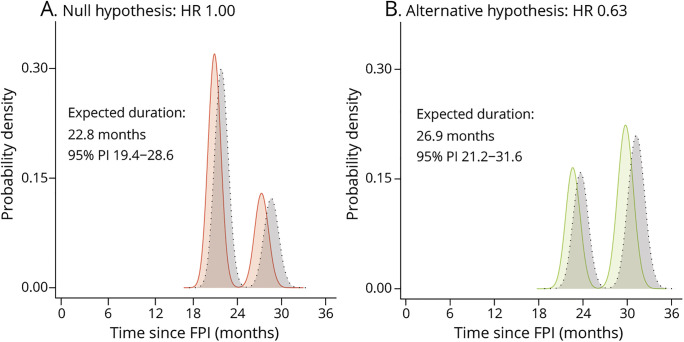
Expected Trial Duration Under the Null and Alternative Hypothesis Distribution of simulated trial duration under the null hypothesis (H_0_, i.e., hazard ratio [HR] 1, panel A) and alternative hypothesis (H_1_, i.e., HR 0.63, panel B). The vertical axis reflects the probability density; its interpretation is similar to a histogram. Due to the lack of a treatment benefit under H_0_, the required number of events occurs more rapidly and the average duration is shorter compared to H_1_. The peaks reflect the average analysis time points; the last peak in panel B is centered around 30 months (i.e., when all assumptions hold). In gray is a scenario when the survival probability in the placebo arm is better than expected (60.0% instead of 56.1%). FPI = first patient first visit; PI = percentile interval of the empirical trial duration ranging from the 2.5th to the 97.5th percentile.

The stopping times of each simulated trial are provided in Figure 2. The peaks reflect the average (interim) analysis time points. The last peak in Figure 2B is centered on the 30 months mark; this reflects the expected trial duration if all assumptions hold, including the treatment effect, and the trial cannot be stopped early. If the treatment is futile compared to placebo (e.g., HR 1), the target number of events is reached sooner and the trial can be stopped earlier. Table 3 provides a comparison with a classical design; the illustrated design results in considerable reductions in sample size, trial duration, total placebo exposure time, and costs. Individual patients, on average, may be exposed longer (+7.4%) to placebo compared to a fixed design when treatment is effective, but shorter when treatment is ineffective (−7.0%). The event-driven design naturally adapts the trial duration to reach the target number of events when one of the design assumptions is inaccurate (e.g., underestimation of the survival probability, illustrated in gray, Figure 2). This underscores the resilience of event-driven designs against misspecification of assumptions and the assurance that power will be attained.

**Table 3:**
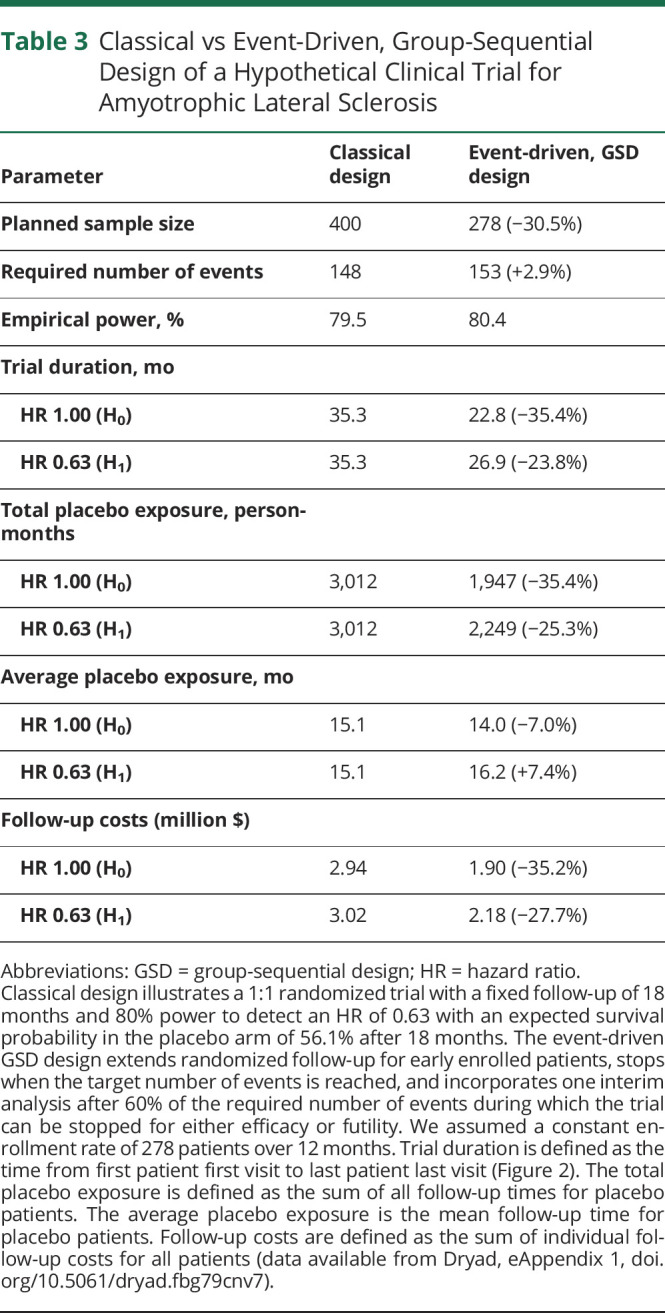
Classical vs Event-Driven, Group-Sequential Design of a Hypothetical Clinical Trial for Amyotrophic Lateral Sclerosis

### Considerations and Refinement After Regulatory Feedback

An effect on only function without an effect on survival has so far not been accepted by the EMA. This may change in the future if the association between survival and function becomes more firmly established (e.g., when a study reveals how a treatment benefit on ALSFRS-R is reflected in life expectancy). For current pivotal studies, it remains therefore critical to obtain adequate information on survival time. Group-sequential methodology results, on average, in smaller and shorter trials, although the number of interim analyses should be justified and not large. A helpful guide could be to determine whether sufficient information on safety, important subgroups, and observational time per patient would be available if a trial is stopped early. To illustrate: if an interim analysis in Figure 2 were performed after 10% of the events, the average follow-up time per patient would only be 4.9 months (vs 14.4 months at 60% of the events). Should a study be stopped early for efficacy, existing safety datasets and means of collecting additional safety data (e.g., utilizing postapproval studies) would have to be discussed with regulatory authorities.

## Discussion

Modifications in the design of clinical trials may not only result in large efficiency gains and reductions in costs, but also widen eligibility criteria and minimize patients' exposure time to ineffective treatments or placebo. Considering the high futility rates in previous ALS clinical trials, and the considerable number of promising treatments, it is critical that the design of future studies is optimized. Our proposed design modifications could provide an important step forwards, where the concepts may serve as a blueprint for future clinical trials with time-to-event endpoints in ALS.

ALS clinical trials are primarily affected by clinical and pathophysiologic heterogeneity, which complicates the detection of treatment effects. Here we quantified the prognostic heterogeneity in clinical trials by using individual risk profiles, and subsequently reduced the observed heterogeneity by using the risk profile as the eligibility criterion.^5,37^ Nevertheless, the risk profile may also be used to study between-trial differences and improve between-study comparability, whereas its distribution can provide insight into the generalizability of trial results and help to define the label for market authorization.^38^ For trial design specifically, risk profiles can be used to improve randomization,^6^ explore risk-based subgroup analyses,^38^ or increase statistical power as a covariate in the final analysis.^39^ Although the risk profile in our study was based on the ENCALS model, the methodology is not limited to a specific model. Continuously optimizing the available prediction rules remains of importance to improve future study design.

As ALS trials are becoming increasingly complex with the arrival of platform trials (e.g., NCT04297683 and NCT04302870) and genotype-targeted studies (e.g., NCT03626012), uncertainty at the design stage may play a progressively more important role. Event-driven or information-based designs may serve as a relatively straightforward strategy to ameliorate the consequences of inaccurate design assumptions, and protect the trial's (or substudies') ability to draw definite conclusions. The main limitation of event-driven designs is the relatively unknown trial duration, both for investigators and patients, and the potentially longer exposure to placebo when enrolled early. This is balanced by the guarantee in statistical power, where simulations can provide extensive insights into the variability between best- and worst-case scenarios and help to refine sample size calculations, also by implementation of interim analyses.

The proposed design can be further refined by integrating clinical outcomes with survival time.^18,40^ These composite endpoints could provide enhanced insights into the expected treatment effect, reduce the effects of (informative) missing data, and may further optimize decision-making about whether to stop a trial early or continue it. Moreover, the position of the regulator may change in the future with a shift towards intermediate endpoints such as ALSFRS-R or respiratory function. Group-sequential designs, extended randomized follow-up, and information-based design may be of similar value in these settings, and a continued dialogue with regulatory bodies will be essential to bring innovation to clinical trials in ALS.

Ultimately, trial design is a dynamic process, where obtaining input from patients, industry, regulators, and funders will be fundamental to the outline of a definite roadmap and to achieving wide-scale adoption. Introducing alternative concepts for the design of clinical trials in ALS may circumvent important pitfalls encountered by classical designs and speed up the development of effective therapy for this debilitating disease.

## Glossary

ALS: amyotrophic lateral sclerosis
ALSFRS-R: ALS Functional Rating Scale–Revised
EMA: European Medicines Agency
ENCALS: European Network for the Cure of ALS
HR: hazard ratio
PRO-ACT: Pooled Resource Open-Access ALS Clinical Trials
VC: vital capacity
